# Fellow Eye in Unilateral Primary Congenital Glaucoma

**DOI:** 10.5005/jp-journals-10008-1217

**Published:** 2017-01-18

**Authors:** Nader HL Bayoumi

**Affiliations:** Associate Professor, Department of Ophthalmology, Faculty of Medicine, Alexandria University, Alexandria, Arab Republic of Egypt

**Keywords:** Fellow, Normal, Primary congenital glaucoma.

## Abstract

**Aim:**

This is a report of the incidence of bilateral cases in a cohort of primary congenital glaucoma (PCG) cases and a study of the biometric characteristics of the fellow normal eyes in unilateral cases.

**Materials and methods:**

The charts of 134 PCG children were reviewed, of which 78 cases (58.2%) were found to have bilateral disease. The remaining 56 patients (41.8%) with unilateral disease had their fellow normal eyes compared with an age-matched cohort of ophthalmologically free children.

**Results:**

There were no differences between the normal fellow eyes of PCG cases and the control eyes in the corneal diameter and central corneal thickness (CCT), whereas the normal fellow eyes of PCG cases had higher intraocular pressure (IOP) and cup/disc (C/D) ratios.

**Conclusion:**

The fellow eyes of apparently unilateral PCG cases are not typically normal anatomically like other children unaffected by PCG.

**Clinical significance:**

A very high index of suspicion has to be kept for PCG cases that present apparently unilaterally, and meticulous prolonged follow-up is mandatory.

**How to cite this article:**

Bayoumi NHL. Fellow Eye in Unilateral Primary Congenital Glaucoma. J Curr Glaucoma Pract 2017;11(1):28-30.

## INTRODUCTION

Primary congenital glaucoma (PCG) is the most common of the childhood glaucomas.^1^ It is a potentially blinding disease, and timely appropriate management is prudent to prevent lifelong blindness. Disappointingly enough, treatment is not always successful and the success rates are variable.^2^ Hence, from the parents, community, and medical standpoints, laterality distribution of the disease is crucial, as well as the characteristics of the fellow -apparently normal - eyes. To the best of the author’s knowledge, there are no published studies comparing the fellow eye in unilateral cases to normal children eyes. The aim of the current study was to report on the lateral-ity distribution of a cohort of PCG cases operated upon in Alexandria Main University Hospital in Alexandria, Egypt, and compare the biometric characteristics of the fellow normal eyes in strictly unilateral PCG cases to a cohort of age-matched normal children eyes.

## MATERIALS AND METHODS

A retrospective chart review of 134 children operated upon for PCG in Alexandria Main University Hospital between March 2005 and October 2012 was conducted. The charts of the PCG children were reviewed to filter out strictly unilateral cases of PCG. Bilateral or unilateral PCG was defined according to the classification system adopted by the childhood glaucoma research network (CGRN).^3^ Given the fact that bilateral PCG may present asymmetrically, strictly unilateral PCG was defined as a child that has demonstrated the classic diagnostic features of PCG in one eye (as defined by the CGRN^3^) and has passed the age limit of 2 years (24 months) and has not presented the clinical features of PCG in the fellow eye, including enlargement of the corneal diameter and/ or axial length, progressive optic nerve cupping, and/ or typical corneal signs of PCG including Haab’s striae, corneal clouding, and/or broad limbus. The clinical characteristics of the fellow normal eyes at around the age of 2 years were reviewed, and this group was designated as “cases.” The age limit of 2 years was chosen as this was the critical timing before which a rise in intraocular pressure (IOP) would invariably result in globe enlargement and the typical features of PCG (buphthalmos), after which a rise in IOP is likely to present in a similar way to adult-onset open-angle glaucoma (without buphthalmos) and would be labeled as late-onset PCG or juvenile open-angle glaucoma (JOAG).^3^

The charts of a cohort of age-matched children were reviewed for biometric characteristics. These children were examined under general anesthesia (GA) on account of the need for an ophthalmic examination when the children were not cooperative enough for an office examination. Records of children that were found to be ophthalmologically free (40 children) only were chosen for comparison and included in the study and were designated as “controls.”

The data were fed into a computer and the statistical workup was conducted using Microsoft Excel (©2007, Microsoft Corporation). Student’s t-test was conducted for comparison of the two groups. A level of significance of 0.05 was chosen, below which results were considered statistically significant.

## RESULTS

The charts of 134 children operated upon for PCG in Alexandria Main University Hospital were reviewed. A total of 56 patients (41.8%) were found to fit the definition of strictly unilateral cases, with a bilaterality incidence of 58.2%. All patients had completed at least 1 year of follow-up and had passed the age limit of 2 years at the time of the last follow-up. The demographic data of the cases and controls are presented in Table 1. There was no statistically significant difference between the two groups in the age at examination (p = 0.206). The clinical and biometric characteristics of the cases and controls are presented in Table 2. There was no statistically significant difference between the two groups in the corneal diameter and central corneal thickness (CCT). The IOP and the cup/disk (C/D) ratio in the cases were statistically significantly higher than in the controls (p = 0.001 and 0.022 respectively).

## DISCUSSION

The current study reports a bilateral PCG incidence of about 60%. This is in accordance with other published reports, as in the studies by Zagora et al^4^ reporting bilateral involvement in 67.1% of PCG cases and the study by Yassin and Al-Tamimi^5^ reporting bilateral involvement in 74% of PCG cases and is in contradistinction to other published reports, as in the study by Aziz et al^6^ reporting bilateral involvement in 99.3% of cases, the study by Tamcelik et al^7^ reporting bilateral involvement in 94.4% of cases, the study by Alanazi et al^8^ reporting bilateral involvement in 82.6% of PCG cases, and the study by Taylor et al^1^ reporting bilateral involvement in 82% of PCG cases. This higher bilaterality incidence in those reports may be related to the disease severity and underlying genetic abnormality in different communities and ethnic groups. The disease is reportedly more severe in consanguineous communities,^2^ and, in these cases, severity may parallel laterality.

**Table 1:** **Table 1:** Demographics of study participants

		*Cases*		*Controls*	
Patients (n, %)		56 (100)		40 (100)	
Males		34 (60.7)		22 (55)	
Females		22 (39.3)		18 (45)	
Eyes (n, %)		56 (100)		40 (100)	
Right		29 (51.8)		20 (50)	
Left		27 (48.2)		20 (50)	
Age at examination (mean ±		25.2 (± 4.3,		33.0 (± 33.7,	
SD, range, median) months		16.1-33.2,		7-144, 25)	
(p = 0.206)		25.3)			

SD: Standard deviation

**Table 2:** **Table 2:** Clinical characteristics of cases and controls

		*Cases*		*Controls*	
IOP (mm Hg), mean (± SD, range, median) (p = 0.001)*		7.3 (± 2.8, 4-16, 6)		5.3 (± 1.8, 2-8, 6)	
Corneal diameter (mm), mean (± SD, range, median) (p = 0.264)		11.9 (± 0.7, 10-13.5, 12)		11.7 (± 0.5, 11-12, 12)	
CCT (|j), mean (± SD, range, median) (p = 0.272)		545.3 (± 36.9, 473-618, 548)		545.2 (± 28.1, 506-623, 539)	
Axial length (mm), mean (± SD, range, median)		22.08 (± 1.47, 19.41-26.9, 22.01)		N/A	
C/D ratio, mean (± SD, range, median) (p = 0.022)*		0.1 (± 0.2, 0-0.8, 0.1)		0.0 (± 0.0, 0-0.1, 0.1)	

*Statistically significant; SD: Standard deviation

The cases and controls in the current report were studied around the age of 2 years and were truly statistically age matched. Surprisingly enough, both the IOP and the C/D ratio were found to be higher in the cases than in the controls. Though statistically significant, this difference was not clinically significant. Given the fact that these eyes were the fellow eyes of PCG cases and were labeled as “normal,” after a significantly long follow-up duration, it would be more appropriate to label them as “currently disease free” rather than normal. After all, whether these differences represent an early stage of what will develop later into JOAG or whether they represent an arrested/abortive form of PCG remains speculative.

It is true that some of the mutations in the CYP1B1 gene - the gene most implicated in PCG - are responsible for JOAG,^9^ and PCG is reported to have variable expressivity and penetrance.^10,11^ This may explain the difference between the fellow eyes and the controls, hypothesizing that these fellow eyes represent arrested or abortive forms of PCG.

Given these findings and given the fact that an elevation of IOP after 2 years of age will not change the biometrics of the eye and will manifest only in optic nerve damage, a very high index of suspicion and a low threshold for the diagnosis of PCG have to be kept for these fellow eyes of initially apparently unilateral cases of PCG.

Limitations of the current study include the lack of important data in the records of controls, such as the axial length and the smaller number of controls than the cases. However, given the antecedent risks of GA, it is prudent to limit its use to “an otherwise impossible to obtain information” and, hence, the limited number of normal children available for inclusion.

In conclusion, PCG may be unilateral in almost 40% of cases, in which the fellow “apparently normal” eyes are different from eyes of otherwise normal children of the same age. Thorough, meticulous, and prolonged follow-up is thus recommended for both eyes of all PCG cases.

## CONCLUSION

The fellow eyes of apparently unilateral PCG cases are not typically normal anatomically like other children unaffected by PCG.

## CLINICAL SIGNIFICANCE

A very high index of suspicion has to be kept for PCG cases that present apparently unilaterally, and meticulous prolonged follow-up is mandatory.
